# Gut bacterial and fungal communities of François’ langur (*Trachypithecus francoisi*) changed coordinate to different seasons

**DOI:** 10.3389/fmicb.2025.1547955

**Published:** 2025-03-05

**Authors:** Jinyuan Liu, Qixian Zou, Diyan Li, Tao Wang, Jialiang Han

**Affiliations:** ^1^School of Pharmacy, Chengdu University, Chengdu, China; ^2^Mayanghe National Nature Reserve Administration, Tongren, China; ^3^School of Basic Medical Sciences, Chengdu University, Chengdu, China; ^4^Office of Academic Affairs, Chengdu University, Chengdu, China

**Keywords:** François’ langur, gut microbiota, seasonal variation, bacterial diversity, fungal diversity, *Akkermansia*, *Cercophora*

## Abstract

**Introduction:**

François’ langur (*Trachypithecus francoisi*), an endangered primate endemic to limestone forests in Vietnam and China, relies on gut microbiota to maintain gastrointestinal stability and adapt to dietary shifts. While gut microbial communities are dynamic and sensitive to seasonal and resource variations, their specific responses in François’ langurs remain poorly characterized. This study investigates seasonal variations in the composition and diversity of gut bacterial and fungal communities in this species to enhance understanding of its ecological adaptations.

**Methods:**

Fresh fecal samples from 22 François’ langurs in Mayanghe National Nature Reserve, China, were collected across four seasons. Bacterial and fungal communities were analyzed using high-throughput sequencing to assess taxonomic composition and α-diversity. Statistical comparisons were conducted to evaluate seasonal differences at phylum and genus levels.

**Results:**

Significant seasonal shifts occurred in both bacterial and fungal communities. Bacterial α-diversity peaked in warmer seasons, whereas fungal diversity was higher in colder months. At the genus level, *Akkermansia* (1.3% relative abundance in summer), a mucin-degrading bacterium linked to gut health, dominated warmer seasons. In contrast, the fungal genus *Cercophora*, associated with plant biomass degradation, was enriched during colder seasons. Seasonal factors strongly influenced microbial structure, with distinct community assemblages observed across all seasons.

**Discussion:**

The inverse diversity patterns of bacterial and fungal communities suggest complementary roles in nutrient extraction under seasonal dietary constraints. *Akkermansia*’s summer prevalence may reflect enhanced mucin utilization during fruit-rich periods, while *Cercophora*’s cold-season dominance likely aids cellulose breakdown in leaf-heavy diets. These dynamics highlight the microbiota’s role in optimizing energy harvest from seasonally variable diets. By elucidating microbial seasonal plasticity, this study provides critical insights for developing conservation strategies tailored to the nutritional ecology of François’ langurs.

## 1 Introduction

The gut microbiota is crucial for maintaining gastrointestinal homeostasis and enabling hosts to adapt to diverse diets (Yang et al., 2022). It supports digestion, nutrient absorption, immune modulation, and metabolism, highlighting its fundamental role in host health. The microbiota’s composition is highly dynamic, changing significantly in response to diet and seasonal shifts (Amato et al., 2015) (Hooper et al., 2012; Markle et al., 2013; Su et al., 2021). Such adaptability aligns microbial functions with host needs, enhancing resilience under varying conditions. Understanding these mechanisms is vital for conserving endangered species (Bergmann et al., 2015; Kartzinel et al., 2019).

Recent studies show seasonal reconfiguration of gut microbiota in various species, such as Hadza hunter-gatherers (Smits et al., 2017), western lowland gorillas and chimpanzees (Hicks et al., 2018), and red squirrels (Ren et al., 2017). These findings emphasize microbiota’s capacity to adapt to environmental and dietary changes, maintaining ecological and physiological balance. Seasonal dietary shifts drive microbial reconfiguration, enabling hosts to adapt to varying conditions. Individual variations in gut microbiota are influenced by seasonal diet and intrinsic factors like gender, especially during non-breeding periods. Understanding this interplay is crucial for developing personalized nutrition strategies and exploring evolutionary pressures shaping microbial communities. Gut microbes are essential for breaking down complex dietary components, facilitating digestion and nutrient extraction in animals. They support ruminants in adapting to plant phenology and physiological demands. Seasonal variation in food resources drives changes in gut microbial composition (Bergmann et al., 2015; Ley et al., 2008). In gregarious animals, coordinated foraging behaviors reduce dietary variation, enhancing collective adaptation to seasonal shifts (Galef and Giraldeau, 2001).

The François’ langur (*Trachypithecus francoisi*) is a primate with a range from northern Vietnam to south-central China (Zhou et al., 2006). Historically, this species was widespread across multiple regions in China. However, over the past three decades, its distribution has significantly contracted due to increasing human activities, including hunting, deforestation, and land use changes (Hu et al., 2004; Huang et al., 2002; Niu et al., 2016). Habitat fragmentation and human disturbance have severely affected François’ langur populations, leading to sharp declines in their (Deng et al., 2019). Over this period, the population has decreased by approximately 2,100 individuals, and the number of isolated distribution points has dropped from 41 to 22 (Hu et al., 2004; Huang et al., 2002). This species primarily consumes leaves, fruits, seeds, bamboo shoots, and occasionally insects, showcasing its dietary flexibility (Hu, 2011). The François’ langur serves as an excellent model for studying gut microbiome plasticity in wild populations. Investigating this species can provide insights into how environmental factors shape gut microbial composition and function, enhancing our understanding of host-microbiome interactions. Despite its ecological significance, studies on the gut microbiota of François’ langurs remain limited.

In this study, we examined the gut microbiota (16S rRNA and ITS) of wild François’ langurs in Yanhe County, Guizhou Province, China. Our objectives were to analyze the structure and diversity of the gut microbiota, identify dominant bacterial and fungal communities, and investigate seasonal variations in gut microbial communities under different environmental conditions.

## 2 Materials and methods

### 2.1 Fecal sample collection

This study was conducted in the Mayanghe National Nature Reserve (MNNR) (28°37′30″–28°54′20″N, 108°3′58″–108°19′45″E), located in northeastern Guizhou Province, southwestern China. The reserve covers an area of 311.13 km area was established in September 1987 to protect the François’ langur and its habitat. The study site, situated at elevations ranging from 280 to 1,441 m, experiences a warm, humid, and rainy climate with distinct seasonal variations in temperature and precipitation. The region encompasses diverse vegetation types, including evergreen broadleaf forests, coniferous forests, mixed coniferous-broadleaf forests, broadleaf forests, bamboo forests, and shrublands. The reserve is home to four wild groups of François’ langurs, comprising a population of 500–600 individuals (Niu et al., 2016).

The 22 François’ langurs included in this study reside in the southwestern portion of the reserve at altitudes between 330 and 760 m. These langurs are well-habituated to human presence. Their diet primarily consists of leaves, fruits, flowers, and buds (Hu, 2011). The langurs typically use caves or cliff platforms as sleeping sites and defecate before departing in the morning. To minimize disturbance, we continuously tracked the langurs during defecation. Prior to sampling, we observed the François’ langurs to identify their sleeping sites and then collected fecal samples under the cliffs the following morning. To avoid sampling duplicates from individual langurs, we ensured that each collection site was separated by more than 3 m. We only collected the middle portion of the feces, which had not been exposed to the external environment. Samples were obtained using sterile gloves and germ-free tools, such as a bamboo skewer. Each sample, weighing 3–5 g, was placed into a labeled sterile collection tube. Using tweezers, we transferred the samples immediately into RNA Later (QIAGEN, Valencia, CA) to preserve RNA stability. The samples were then stored at −80°C in an ultralow temperature freezer for later analysis. This study took place in October 2023, and January, May, and July 2024, during which we collected a total of 22 fecal samples (Supplementary Table S1).

### 2.2 Ethics approval

All fecal sampling was conducted with permission from the Administration of MNNR (202410). No invasive collection of animal tissues that could cause harm or distress to the animals was performed. Fecal samples were only collected after the François’ langurs had left their sleeping sites to minimize any potential stress.

### 2.3 DNA extraction, PCR amplification, and 16S rRNA sequencing

We used a Zymo Research BIOMICS DNA Microprep Kit (Cat# D4301) to extract total genomic DNA (gDNA) from the feces of François’ langurs. The integrity of the gDNA was assessed via 0.8% agarose gel electrophoresis. Nucleic acid concentration and purity were measured using the Tecan F200 and the PicoGreen dye method. For bacterial community analysis, we amplified the V4 region of the 16S rRNA gene by PCR using primers 515F (5′-GTGYCAGCMGCCGCGGTAA-3′) and 806R (5′-GGACTACHVGGGTWTCTAAT-3′). For fungal community analysis, we amplified the fungal ITS2 region using primers ITS3 (5′-GATGAAGAACGYAGYRAA-3′) and ITS4 (5′-TCCTCCGCTTATTGATATGC-3′). The PCR reaction was performed in a 50 μL volume, consisting of 5 μL 10 × PCR buffer for KOD-Plus-Neo, 5 μL 2 mM dNTPs, 1.5 μL of each forward and reverse primer (5 μM), 1 μL KOD-Plus-Neo DNA polymerase (1 U/μL), and 2 μL sample DNA, with the remainder filled with ddH_2_O. We included ultrapure water as a negative control in each PCR amplification to prevent false-positive results. The PCR amplification conditions were as follows: an initial denaturation at 94°C for 1 min, followed by 25–30 cycles of 94°C for 20 s (denaturation), 54°C for 30 s (annealing), and 72°C for 30 s (extension), with a final extension at 72°C for 5 min. The PCR products were mixed with a six-fold loading buffer and analyzed by electrophoresis on a 2% agarose gel. The gel-purified products were recovered using a Zymoclean Gel Recovery Kit (D4008). We quantified the recovered PCR products using 2% agarose gel electrophoresis and a Qubit^®^ 2.0 Fluorometer (Thermo Scientific). Each sample included three PCR replicates. Equal amounts of PCR products from the linear phase were pooled for library construction. We used a NEBNext Ultra II DNA Library Prep Kit for Illumina (New England BioLabs) to prepare the library, following the manufacturer’s instructions. The purified PCR-amplified fragments were sequenced using the PE250 sequencing method on the Illumina NovaSeq 6000 with a SP Reagent Kit V1.5.

### 2.4 16S rRNA and ITS-seq quality control

We merged paired-end sequences using FLASH and extracted sample-specific sequences from the raw reads using the sabre tool, trimming the barcode sequences. Quality control was performed using QIIME2 (Bolyen et al., 2019), applying the following criteria: sequences with an average quality score below 30 were excluded, sequences shorter than 200 base pairs were removed, and sequences containing more than 0 ambiguous nucleotides (N) were discarded.

### 2.5 Operational taxonomic unit cluster and species annotation

The remaining high-quality sequences were analyzed to generate operational taxonomic units (OTUs) using Uparse software (version 7.0.1001) with a 97% similarity threshold (Edgar, 2013). OTUs that appeared only once across all samples and did not align with the reference database were excluded from further analysis. After clustering across all 16 François’ langurs’ samples and removing singleton OTUs, a total of 3,154 OTUs were identified. Nevertheless, many of these OTUs were rare, appearing only in a limited number of samples.

Taxonomic annotations for each OTU, including classification to the levels of kingdom, phylum, class, order, family, genus, and species, were assigned using the SSUrRNA library from the Silva database (Quast et al., 2013).^1^ The Mothur algorithm (Kozich et al., 2013) was applied to perform these annotations. To assess phylogenetic relationships among different OTUs and dominant species within the samples or groups, multiple sequence alignments were performed using MUSCLE software (version 3.8.31) (Edgar, 2004). Furthermore, the abundance of OTUs was normalized to the sample with the minimum sequence count to ensure comparability across samples.

### 2.6 α-diversity and β-diversity analysis

We analyzed species diversity complexity using α-diversity, calculated from the normalized OTUs with four indices: Simpson, Shannon, Chao1, and PD. These analyses were conducted using QIIME2 software (Bolyen et al., 2019). Among these indices, Shannon and Simpson were employed to assess community diversity, Chao1 was used to evaluate community richness, and PD was utilized to determine the evenness of species distribution. To identify differences in α-diversity indices across the four seasons, the Wilcoxon rank-sum test was performed using R software. Additionally, β-diversity analyses were performed to assess differences between samples. These analyses involved calculating β-diversity using both BC distances and weighted/unweighted UniFrac distances through QIIME2 software (Bolyen et al., 2019). The BC ordination analysis provided position values along an ordination axis and the distances of the samples from this axis, reflecting differences in community composition.

### 2.7 Principal coordinate analysis

We performed Principal Coordinate Analysis (PCoA) to identify principal coordinates and visualize complex, high-dimensional datasets. The distance matrix, previously calculated using weighted and unweighted UniFrac distances, was transformed into a new set of orthogonal axes. The first principal coordinate accounted for the largest variation among samples, while the second principal coordinate explained the second largest variation, and so on. We conducted the PCoA analysis using the WGCNA package (Langfelder and Horvath, 2008) in conjunction with the stat and ggplot2 packages within the R software environment.

### 2.8 Prediction of the functional profiles of microbial communities

We used PICRUSt2 to predict the functional profiles of gut bacterial communities across different seasons (Douglas et al., 2019). To further infer the metabolic and ecological functions of these communities, we employed FAPROTAX (Functional Annotation of Prokaryotic Taxa). For the fungal community, we predicted functional profiles using FUNGuild (Fungi Functional Guild) (Nguyen et al., 2016).

### 2.9 Community difference analysis

We conducted pairwise comparisons between sites to assess community differences using ANOSIM (Analysis of Similarities). This analysis, based on Bray-Curtis (BC) ordination, employed 10,000 permutations to evaluate the validity of group divisions.

## 3 Results

### 3.1 Gut bacterial and fungal composition of François’ langur in different seasons

**FIGURE 1:**
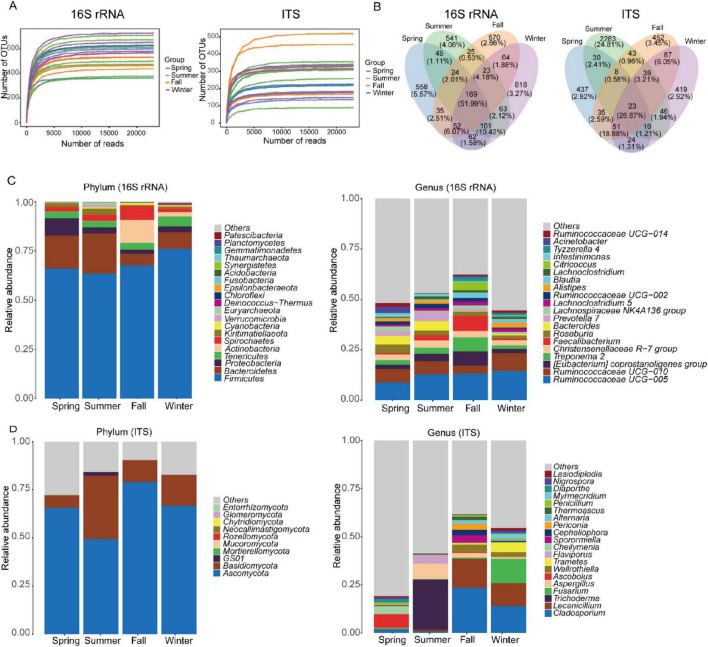
Characterization of gut microbial communities in François’ langur: **(A)** rarefaction curves reflecting the diversity of bacterial and fungal OTUs from François’ langur gut. **(B)** Venn diagram illustrating the overlap of OTUs across different seasons, with numbers indicating the counts of OTUs unique to each section. Bar charts depicting the relative abundance of dominant gut bacterial **(C)** and fungal **(D)** species at the phylum and genus levels.

We obtained 741,552 high-quality clean reads (33,706 reads per sample) from 16S rRNA sequencing and 745,860 high-quality clean reads (99,902 reads per sample) from ITS sequencing. Rarefaction curves for the sobs and Shannon indices at the OTU level plateaued as sequencing depth increased, indicating sufficient sequencing coverage (Figure 1A). These results confirmed that each fecal sample contained enough OTUs to capture the maximum bacterial and fungal diversity. For the gut bacterial community, we classified the 3,154 OTUs into 794 species, 666 genera, 291 families, 166 orders, 73 classes, and 32 phyla. Among these, 169 OTUs overlapped across the four seasons, accounting for 51.99% of the total relative abundance (Figure 1B). For the gut fungal community, we classified the 3,987 OTUs into 608 species, 456 genera, 262 families, 103 orders, 40 classes, and 11 phyla. Of these, 23 OTUs overlapped across the four seasons, representing 26.87% of the total relative abundance (Figure 1B).

At the phylum level, the gut bacterial community of François’ langur displayed seasonal variation. Firmicutes, Bacteroidetes, and Proteobacteria were the dominant phyla in spring and summer. In fall, Firmicutes, Actinobacteria, and Spirochaetes were most prevalent, while in winter, Firmicutes, Bacteroidetes, and Tenericutes dominated (Figure 1C). At the genus level, the composition also varied by season. In spring, summer, and winter, *Ruminococcaceae*, *Lachnospiraceae*, and the *Clostridiales vadinBB60* group were the predominant taxa. However, in fall, the dominant genera included *Ruminococcaceae*, *Lachnospiraceae*, and *Spirochaetaceae* (Figure 1C). The top20 genera level bacteria composition of each sample was analyzed in four seasons (Supplementary Figure S1A). In contrast, the gut fungal community of François’ langur showed no seasonal variation at the phylum level, with Ascomycota and Basidiomycota remaining consistently dominant across all seasons (Figure 1D). At the genus level, however, the fungal community exhibited marked seasonal shifts. *Ascobolus* and *Cheilymenia* were the most prevalent genera in spring. During summer, *Trichoderma* and *Aspergillus* dominated. In fall, *Cladosporium*, *Lecanicillium*, and *Wallrothiella* were the leading genera, while in winter, *Cladosporium*, *Fusarium*, and *Lecanicillium* prevailed (Figure 1D). The fungal composition at the genus level for the top 20 genera in each sample was analyzed across four seasons (Supplementary Figure S1B).

### 3.2 Dynamic change of gut bacterial and fungal communities of François’ langur in different seasons

**FIGURE 2:**
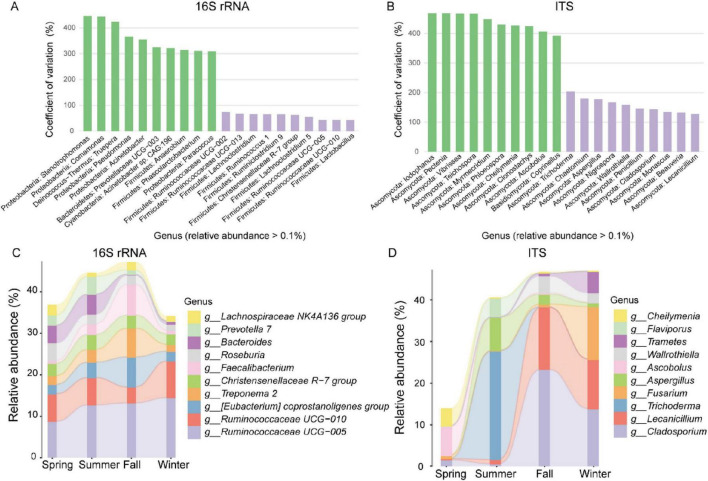
Seasonal variations in gut microbiota of François’ langur. Variation coefficients of relative abundance of bacteria **(A)** and fungal **(B)** at the genus level. Green represents the ten genera with the greatest significant variation coefficients at the genus level, and purple represents the ten genera with the least. Sankey map showing the relative abundances of bacteria **(C)** and fungal **(D)** at the genus level over the four seasons.

To identify genera with the most significant abundance fluctuations across the four seasons, we used the Coefficient of Variation (CV) to measure variability in bacterial and fungal genus abundance. For the bacterial community, *Stenotrophomonas*, *Comamonas*, and *Truepera* exhibited the highest CV values, indicating the largest fluctuations, while *Lactobacillus*, *Ruminococcaceae UCG-010*, and *Ruminococcaceae UCG-005* showed the smallest CV values, indicating the least variability (Figure 2A). In the fungal community, *Iodophanus, Pectenia*, and *Vibrissea* had the highest CV values, whereas *Lecanicillium*, *Beauveria*, and *Monascus* displayed the lowest (Figure 2B).

We analyzed the community composition using a Sankey map to investigate the seasonal dynamics of gut bacterial and fungal microorganisms in François’ langur. For the bacterial community, *Ruminococcaceae UCG-005* showed a consistent upward trend across all seasons. The relative abundances of (*Eubacterium*) *coprostanoligenes group*, *Treponema 2*, and *Faecalibacterium* increased notably in fall, while Bacteroides experienced a sharp decline during the same season (Figure 2C). In the fungal community, seasonal dynamics were pronounced. *Trichoderma*, *Aspergillus*, and *Flaviporus* increased substantially in summer but declined sharply in fall and winter. Conversely, *Cladosporium*, *Lecanicillium*, and *Wallrothiella* exhibited significant increases during fall (Figure 2D).

### 3.3 Seasonal variation in α diversity

We conducted α-diversity analysis based on sequencing depth with a mean Good’s coverage of 99.94% (range: 99.68–99.98%) for the gut bacterial community of François’ langurs across different seasons. The analysis incorporated key α-diversity metrics, including the Shannon index, Simpson index, Chao 1, and Faith’s Phylogenetic Diversity (PD). Linear mixed models were employed to examine differences in gut microbial community diversity across seasons.

**FIGURE 3:**
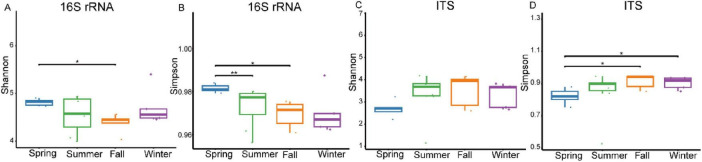
Box plots illustrating species richness within samples, which represent seasonal fluctuations in the α diversity of the gut bacteria **(A,B)** and fungal **(C,D)** of François’ langurs. Richness is measured by the Shannon and Simpson indexes. *P < 0.05 (Wilcoxon rank-sum test), **P < 0.01.

The results revealed significant seasonal variation in the gut bacterial community α-diversity of François’ langurs. Analysis based on OTUs demonstrated that the Shannon diversity index exhibited significant seasonal fluctuations. Specifically, species diversity was higher in spring samples compared to fall samples (Figure 3A). Similarly, the Simpson diversity index showed seasonal differences, with spring samples exhibiting higher species diversity than those from summer and fall (Figure 3B). The Chao 1 index also showed seasonal variability, with winter samples demonstrating greater species diversity than summer samples (Supplementary Figure S2A). Additionally, the PD index exhibited seasonal shifts, with winter samples showing a higher level of species diversity composition compared to spring samples (Supplementary Figure S2B).

In the gut fungal community analysis, α-diversity was relatively lower during spring across all four diversity indices. Analysis based on OTUs revealed that the Shannon diversity index showed no significant differences across the four seasons (Figure 3C). However, seasonal differences were evident in the Simpson diversity index, with fall and winter samples exhibiting higher species diversity compared to spring samples (Figure 3D). Seasonal variability was also observed in the Chao 1 index, with summer samples showing higher species diversity than spring and winter samples (Supplementary Figure S2C). Furthermore, the PD index exhibited seasonal variation, with summer samples displaying greater species diversity composition compared to other seasons, particularly spring (Supplementary Figure S2D).

### 3.4 Seasonal variation in β diversity

**FIGURE 4:**
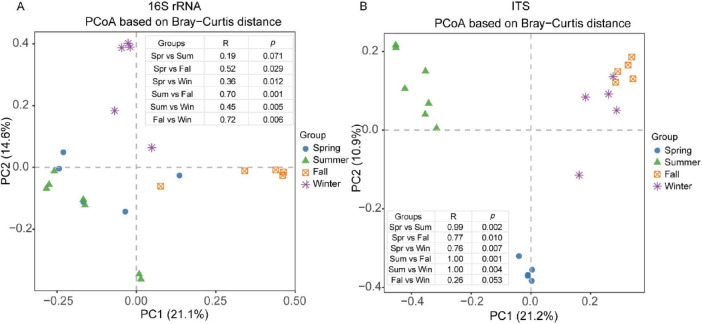
PCoA analysis of the composition of the gut microbial of François’ langurs in different seasons. PCoA score plot based on Bray–Curtis distance analysis among the four seasons for bacterial **(A)** and fungal **(B)**.

Below is the extensively edited and polished manuscript section. The revisions ensure clarity, proper grammar, concise academic writing, and adherence to the required format while retaining all necessary details, figure references, and citations. We performed PCoA to explore sample-to-sample differences and identify patterns across seasons using commonly applied distance metrics. For the gut bacterial community, we compared β-diversity measures, including weighted and unweighted UniFrac distances and Bray–Curtis distances, across different geographical populations. The PCoA score plot revealed a clear separation of samples collected in spring, summer, fall, and winter (Figure 4A), except for the comparison between spring and summer, which showed no significant difference (*P* = 0.071). Analysis of unweighted UniFrac and weighted distances confirmed similar patterns across different seasons (Supplementary Figure S3A). Specifically, PCoA based on the weighted UniFrac distance indicated similarity between spring and summer (*P* > 0.05, *R* = 0.17), while the other seasons exhibited statistically significant separation (*P* < 0.05) (Supplementary Figure S3B).

For the gut fungal community, the PCoA score plot based on Bray–Curtis distance showed a notable similarity between fall and winter (*P* > 0.05, *R* = 0.26). Conversely, other seasons displayed distinct separations, as confirmed by ANOSIM, which highlighted these differences (*P* < 0.05, R > 0.75) (Figure 4B). Additionally, PCoA analysis using unweighted UniFrac distance demonstrated a clear distinction between samples collected in spring and those from other seasons (Supplementary Figure S3C). Weighted UniFrac distance analysis showed significant dissimilarity between spring and summer and between fall and winter, although all R values remained below 0.25 (Supplementary Figure S3D).

### 3.5 Season-associated microorganisms in the bacterial and fungal community of François’ langur

**FIGURE 5:**
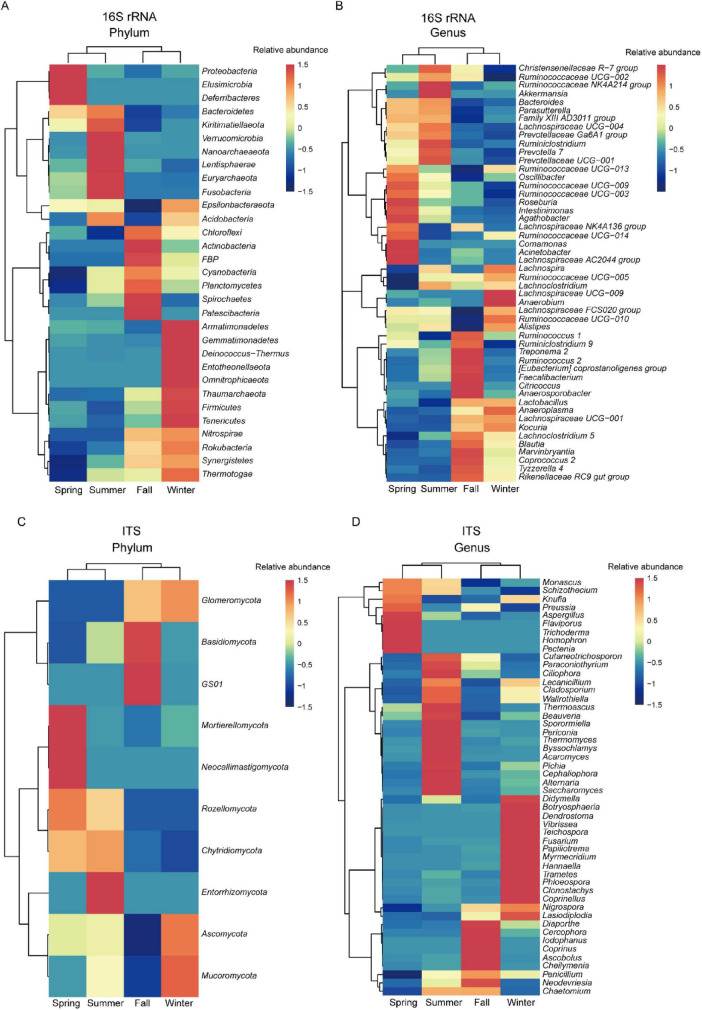
Heatmap illustrating LEfSe test results (with LDA scores > 3 and *P* < 0.05) for the phylum and genus levels of gut microbiota in François’ langur across the four seasons. Heatmap displays bacteria at the phylum **(A)** and genus **(B)** levels, as well as fungal at the phylum **(C)** and genus **(D)** levels.

To further explore seasonal variations in gut microbiota composition, we conducted a Linear Discriminant Analysis Effect Size (LEfSe) analysis (LDA > 3, *P* < 0.05) on the relative abundances of bacterial communities at both the phylum and genus levels across different seasons in François’ langurs. At the phylum level, the analysis revealed that Proteobacteria, Elusimicrobia, and Deferribacteres exhibited significantly higher abundances in the gut microbiota during spring compared to other seasons. Verrucomicrobia, Nanoarchaeaeota, and Euryarchaeota were significantly more abundant in the gut bacterial community during summer than in other seasons, indicating their strong seasonal indicator effect. During the fall, Actinobacteria, Spirochaetes, and Patescibacteria showed significantly greater abundance in the gut microbiota than during other seasons. Conversely, Armatimonadetes, Gemmatimonadetes, and Deinococcus-Thermus were substantially more abundant in winter than in other seasons (Figure 5A). At the genus level, *Acinetobacter*, *Comamonas*, and *Agathobacter* were significantly more abundant in spring compared to the other seasons. In summer, the gut bacterial communities of François’ langurs showed elevated abundances of *Ruminococcaceae NK4A214 group* and *Akkermansia*, with both exhibiting significant season indicator effects. During the fall, *Treponema 2* and *Ruminococcus 2* were significantly more abundant than in other seasons. In contrast, *Lachnospiraceae UCG−009* and *Anaerobium* were substantially more abundant during the winter months compared to other seasons (Figure 5B).

These findings provide insights into how gut bacterial community composition varies across seasons in François’ langurs, with specific phyla and genera exhibiting distinct seasonal patterns. To examine the seasonal variations in fungal community composition and highlight differences among microbial groups, we utilized heatmaps for a comprehensive community composition analysis. At the phylum level, Mortierellomycota and Neocallimastigomycota were significantly more abundant in spring compared to other seasons. During summer, the abundance of Entorrhizomycota was notably higher in the gut fungal community of François’ langurs compared to other seasons, showing a significant season indicator effect. In the fall, Basidiomycota and GS01 exhibited significantly greater abundances in the gut microbiota than during other seasons. Conversely, Mucoromycota was substantially more abundant during winter than in the other seasons (Figure 5C). At the genus level, *Trichoderma*, *Homophron*, and *Pectenia* demonstrated significantly higher abundances *in spring* compared to the other seasons. In summer, the gut fungal community was characterized by higher abundances of *Thermomyces*, *Byssochlamys*, and *Acaromyces*, with these genera exhibiting significant season indicator effects. During the fall, *Cercophora*, *Iodophanus*, and *Coprinus* showed significantly higher abundances in comparison to other seasons. Meanwhile, *Fusarium*, *Papiliotrema*, and Myrmecridium were considerably more abundant during winter than in other seasons (Figure 5D).

### 3.6 Predicted function of gut bacterial and fungal community of François’ langur

**FIGURE 6:**
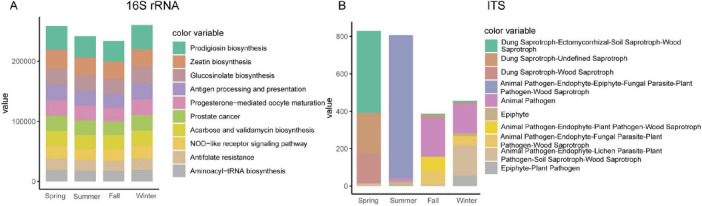
Functional predictions of bacteria **(A)** and fungal **(B)** using 16S rRNA and ITS results combined with genomic databases.

To assess the functional potential of the gut bacterial community, we analyzed the 16S rRNA sequencing data in combination with genomic databases to infer metagenomic profiles. The analysis using PICRUSt2 revealed no significant functional differences across the four seasons. The predominant functional pathways identified included Prodigiosin biosynthesis, Zeatin biosynthesis, and Glucosinolate biosynthesis (Figure 6A). To evaluate the functional capabilities of the gut fungal community, we employed FUNGuild (Fungi Functional Guild) to establish a database linking fungal taxonomy to functional guilds. Using this database for functional classification, we identified significant seasonal variations in the functional composition of the gut fungal community. In spring, the Dung Saprotroph-Ectomycorrhizal-Soil Saprotroph-Wood Saprotroph functional guild was dominant. During summer, the functional composition was primarily characterized by Animal Pathogen-Endophyte-Epiphyte-Fungal Parasite-Plant Pathogen-Wood Saprotroph. In autumn, Animal Pathogen was the leading functional group. In winter, both Animal Pathogen and a composite functional group comprising Animal Pathogen-Endophyte-Lichen Parasite-Plant Pathogen-Soil Saprotroph-Wood Saprotroph were dominant (Figure 6B).

## 4 Discussion

Gut microbes participate in a mutually beneficial symbiotic relationship with their host, modulating the host’s gastrointestinal immune system while reflecting evolutionary adaptations at both the host and microbial cellular levels (Geva-Zatorsky et al., 2017; Lee and Mazmanian, 2010; Ley et al., 2006). This study conducted a comprehensive and systematic investigation of seasonal variations in the gut bacterial and fungal communities of captive François’ langurs. The study found that the diversity of gut microbiota in several rare and protected animals increases during the summer, including the giant panda and plateau pika (Huang et al., 2023; Ren et al., 2024). The analysis identified three dominant bacterial genera in the spring gut microbiota of François’ langurs: *Acinetobacter*, *Comamonas*, and *Agathobacter*. *Acinetobacter* has been shown to degrade various organic contaminants and produce antimicrobial agents that inhibit the growth of plant pathogens (Dahal et al., 2023). This ability is particularly relevant in the context of the langurs’ diet, which includes a variety of plant materials. The presence of Acinetobacter may help the langurs to mitigate the potential harmful effects of plant secondary compounds, thereby facilitating a broader dietary intake. *Comamonas*, another dominant genus in spring, exhibits the ability to dynamically adjust its biofilm-forming behavior, allowing it to adapt to fluctuating environmental conditions (Wu et al., 2019). This adaptability is crucial for the langurs as they navigate seasonal changes in food availability and quality. *Agathobacter*, which produces short-chain fatty acids (SCFAs) with recognized anti-inflammatory properties, stimulates immune responses and contributes to homeostasis in the host (Abdugheni et al., 2022). The presence of Agathobacter in spring may be particularly beneficial as the langurs’ immune systems are challenged by the transition from a winter diet to a more diverse spring diet. During the summer, the dominant gut bacterial genera in François’ langurs were the *Ruminococcaceae NK4A214 group* and *Akkermansia*. The *Ruminococcaceae NK4A214* group is recognized for its ability to degrade cellulose and generate beneficial metabolites that enable the host to derive energy from dietary fiber (Du et al., 2023). This is particularly relevant in summer when the langurs’ diet is rich in fibrous plant materials. *Akkermansia* is a well-known mucin degrader and is classified within the Verrucomicrobiota, representing the sole known member of this phylum to inhabit vertebrate guts (González et al., 2023). The presence of *Akkermansia* in summer may be linked to the langurs’ increased consumption of mucin-rich foods, such as fruits and flowers, which are abundant during this season. In the fall, the abundance of *Treponema 2* and *Ruminococcus 2* in the gut microbiota significantly increases. *Treponema 2* generates ATP through a unique combination of glycolytic and pyruvate fermentation pathways, alongside the putative Rnf complex and ATP synthase (Radolf et al., 2016). This metabolic versatility allows *Treponema 2* to efficiently utilize a range of substrates, which is particularly advantageous in the fall when the langurs’ diet shifts to include more energy-dense foods. *Ruminococcus 2* converts dietary fiber into SCFAs, such as acetate, propionate, and butyrate (Juge, 2023). In winter, the abundance of *Lachnospiraceae UCG-009* and *Anaerobium* also rises significantly. *Lachnospiraceae UCG-009* is a well-known SCFA producer and may influence host metabolism, including the regulation of lipid and blood sugar levels, through its metabolic byproducts (Vacca et al., 2020). The presence of *Lachnospiraceae UCG-009* in winter may be particularly important as the langurs’ diet is limited to less nutritious foods. *Anaerobium* comprises strictly anaerobic bacteria that assist in breaking down undigested food residues in the gut (Stouthamer and Schink, 1994). By interacting with gut epithelial and immune cells, Anaerobium regulates the host’s immune response and maintains intestinal immune homeostasis (Maier et al., 2014).

In the fungal community, *Trichoderma*, *Homophron*, and *Pectenia* were notably more prevalent in the spring. *Trichoderma*, known for producing antibiotics that inhibit the growth of other microorganisms, plays an anti-infective role in both human and plant microenvironments (Schuster and Schmoll, 2010). The presence of *Trichoderma* in spring may help the langurs to combat potential pathogens as they transition to a more diverse diet. *Homophron* may regulate intercellular connections (Maruyama and Kitamoto, 2019), which could be beneficial in maintaining the structural integrity of the gut microbiota. *Pectenia* secretes pectinases, enzymes that break down pectin and contribute to the decomposition of plant polysaccharides (Atta and Ruiz-Larrea, 2022). The presence of *Pectenia* in spring may facilitate the breakdown of pectin-rich plant materials, which are abundant in the langurs’ diet during this season. In the summer, the gut fungal community showed a significant increase in *Thermomyces*, *Byssochlamys*, and *Acaromyces*. *Thermomyces* is a thermotolerant fungus that decomposes plant biomass, particularly hemicellulose (Zhang et al., 2015). *Byssochlamys* not only breaks down plant matter but also produces mycotoxins that pose potential health risks to humans and animals (Puel et al., 2005). The presence of *Byssochlamys* in the langurs’ gut microbiota may reflect a trade-off between the benefits of efficient plant material breakdown and the risks of mycotoxin exposure. In the fall, the abundance of *Cercophora* increased significantly. *Cercophora* fungi degrade plant biomass, especially cellulose, and may aid in breaking down plant food in the animal gut, helping the host extract energy from it (Doveri, 2016). The presence of *Cercophora* in fall may be particularly beneficial as the langurs’ diet shifts to include more energy-dense plant materials. In winter, *Fusarium* and *Papiliotrema* became significantly more abundant. Fusarium species are known pathogens of animals and can cause diseases in plants, humans, and animals (Armer et al., 2024). *Papiliotrema* has potential applications in biotechnology, particularly in single-cell oil production (de Almeida et al., 2022).

## 5 Conclusion

Our study presents an in-depth analysis of the gut microbiota in François’ langurs, underscoring the intricate relationship between seasonal fluctuations and microbial ecosystems. We observed substantial variations in the diversity, composition, and predicted functionality of the microbiota throughout the year. Our findings indicate that the seasonal transitions in the langurs’ gut microbiota play a pivotal role in their health and nutritional status, overshadowing the impact of host genetics on microbial makeup. A significant component of the langurs’ health variability is tied to the seasonal shifts in their gut microbiota. It is worth noting that certain bacterial phyla, including Firmicutes and Proteobacteria, along with fungal groups such as Ascomycota and Basidiomycota, were closely linked to seasonal adjustments. This association suggests their crucial role in bolstering the langurs’ capacity to adapt to environmental changes and optimize their metabolic processes. These discoveries provide fresh insights into the multifaceted roles of gut microbiota in wild primates and could inform the development of targeted conservation efforts for François’ langurs.

## Data Availability

The data in this study have been deposited in the NCBI under accession number PRJNA1196118.

## Footnotes

1
https://www.arb-silva.de/
