# Supplementary figures and images for: Gut bacterial and fungal communities of François’ langur (Trachypithecus francoisi) changed coordinate to different seasons
Source: Front Microbiol. 2025 Mar 5;16:1547955. doi: 10.3389/fmicb.2025.1547955 (PMC11920163; doi:10.3389/fmicb.2025.1547955)

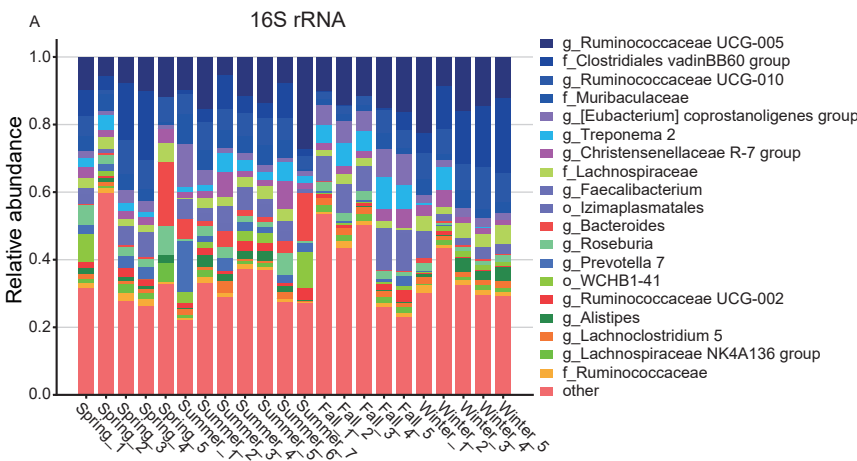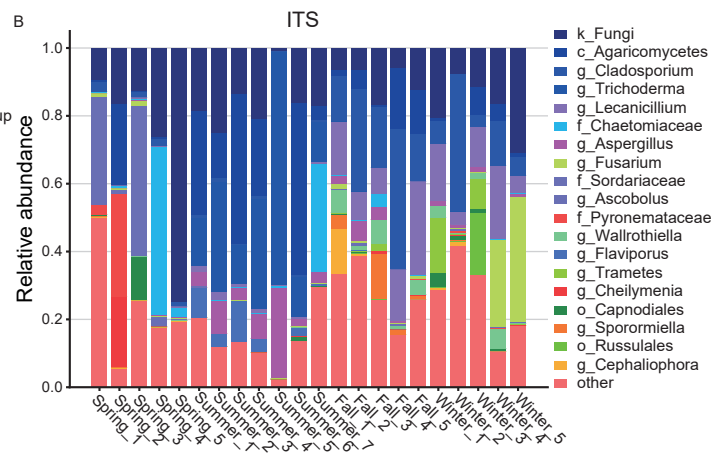

Supplement: Supplementary file 2 [file Data_Sheet_1.pdf]

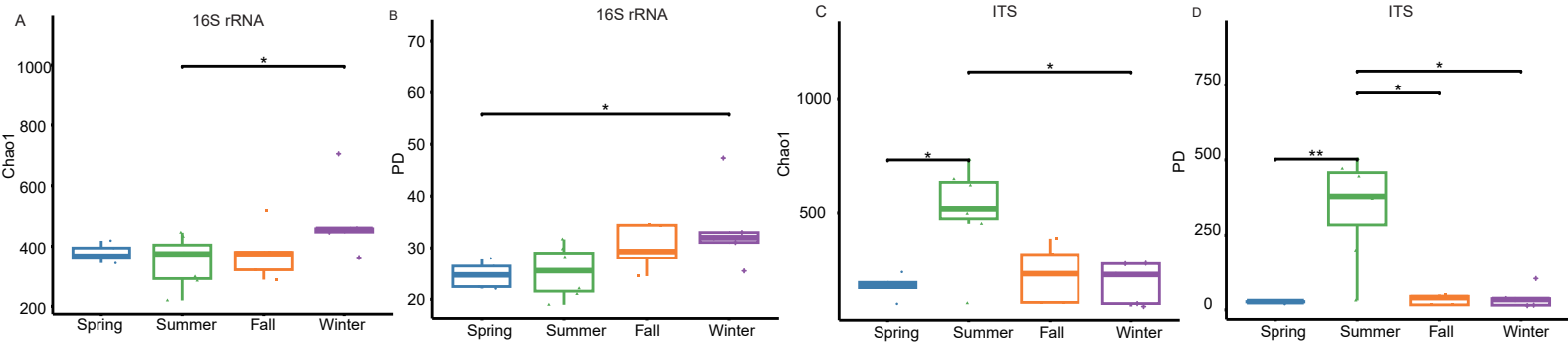

Supplement: Supplementary file 3 [file Data_Sheet_2.pdf]

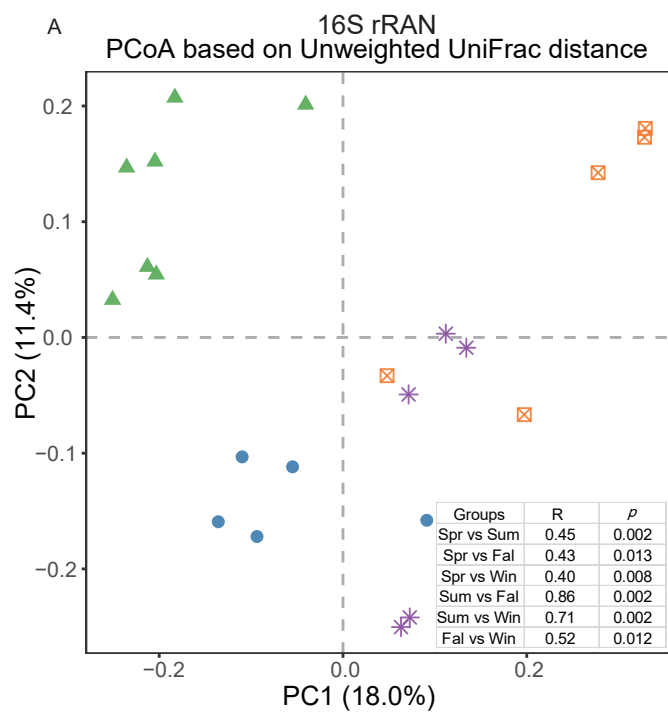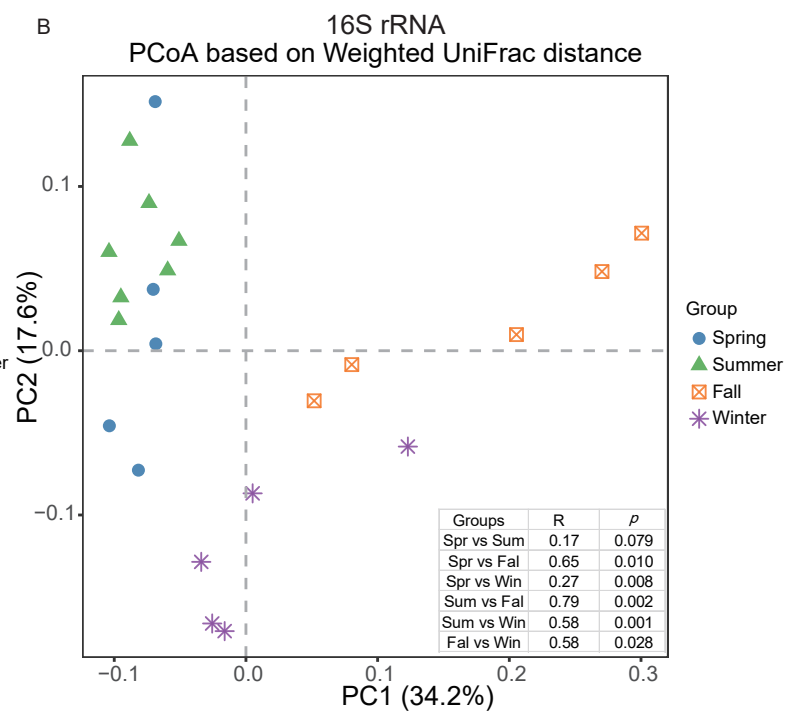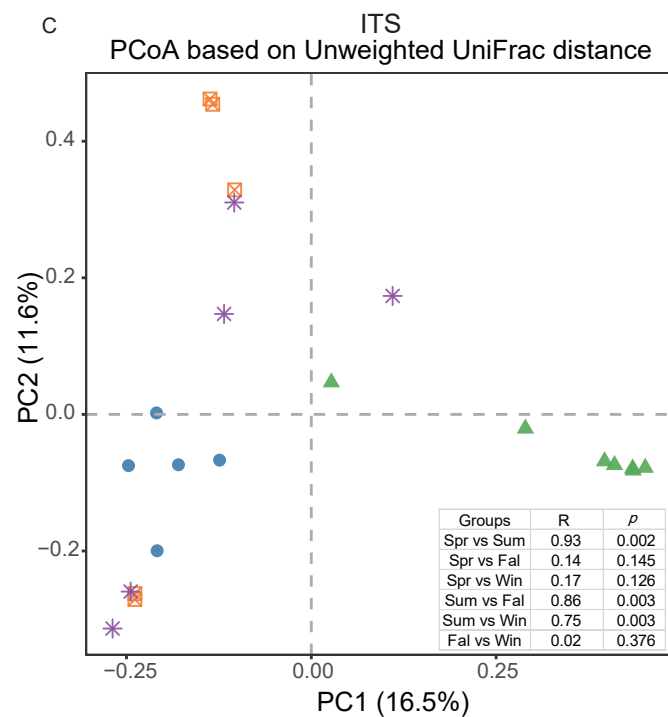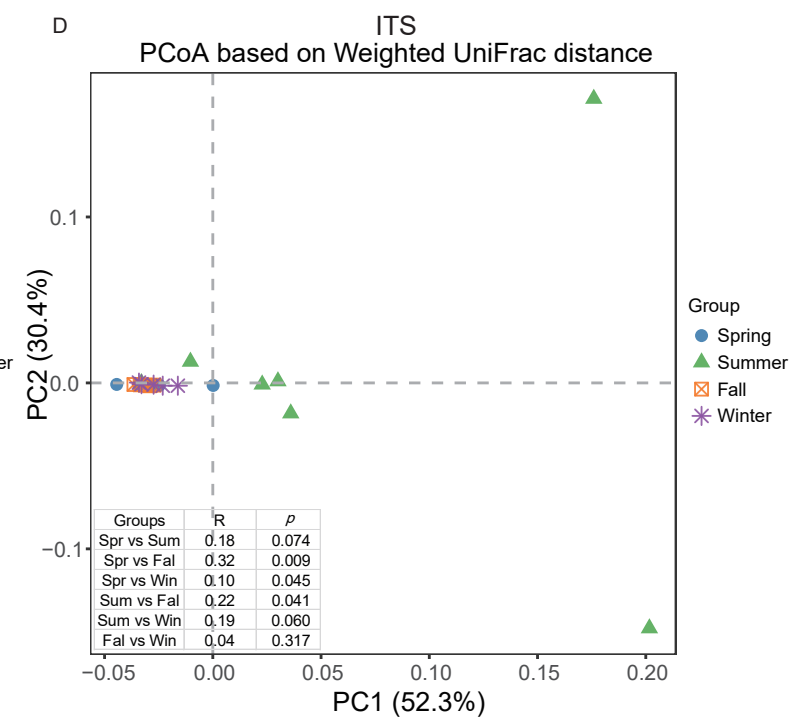

Supplement: Supplementary file 4 [file Data_Sheet_3.pdf]
